# Turning by External Manipulation

**Published:** 1861-08

**Authors:** 


*Turning, by External Manipulation.*—The principal advantages claimed for this operation by Dr. Noeggerath, *New York Jour. of Med., Nov.* 1859, are, its safety to mother and child, dispensing with turning by the feet, and bringing the child in the safest presentation, without impairing the chances of success by internal manipulation in case of failure by this external process.

The operation is indicated in all those cases where the child presents an oblique or transverse position, in such a way that the head is situated not far from the pelvic entrance, provided that there is nothing in the case which actually demands, or may possibly after a while demand, an actual interference for hastening the process of labor. For example, in all cases of neck and shoulder presentations, in all presentations of the trunk where the head is situated nearer to the pelvis than to the chest, we may try to correct the position of the child by external manipulations. The caution not to try version by external manipulation, in cases where we want a prompt delivery of the child by all means, is derived from the fact that such trials do sometimes prove unsuccessful, so at least, unless continued for a long while. It would be wrong, for instance, to try these manipulations in a case of hemorrhage or convulsions, because of the loss of valuable time, which ought to have been applied in the performance of a prompter mode of delivery; and even should we succeed in a similar instance to bring the head down to the upper straight, this would afterward prove a bar to the easy performance of delivery by the feet, if required by insufficiency of pains which failed to firmly engage the head in the pelvic brim, as a preparatory step for delivery with the forceps.

The correct diagnosis of the situation of the fœtus in utero, by external examination, is the preparatory step, and an intrinsic part of the operation. Before attempting to perform it, the operator must have in every single case a distinct idea of the presentation of the child in his mind, to the confirmation of which repeated inspection, palpitation and auscultation must be called to aid; internal examination will, in the great majority of cases, yield only negative results. This done, the woman is ordered to lie on her back, while the physician takes his position on the side of the bed opposite that where the head is located. Suppose the head is felt in the *left* iliac fossa, the operator places his *right hand* upon the cranial protuberance, while his left hand is placed on that portion of the uterus where the nates are situated. Now, gentle friction are made upon the points indicated, and at once a pressure effected on the head with a tendency to push it downward and toward the mesian line, while the breech is gently pushed upward and toward the opposite side. All this is done during an interval of the pains. As soon as another pain begins, both hands keep their place, and the woman is ordered to turn on her left side. With the remission of the pain the same mnnœuvre must be repeated, and continued until a change of presentation is effected. This having been ascertained by internal examination, the woman has to continue the posture on her left side, and a small hard pillow is to be placed just underneath that portion of the abdomen where the fœtal head was at first situated. If after a number of pains the head is found to have retaken its former situation, the manipulations must be repeated, and after turning has been effected again, it is advisable to rupture the membranes, in order to keep the head from returning to where it was formerly imbedded. The rest of labor is conducted like an ordinary vertex presentation.

In some cases it is necessary to repeat these manipulations three or four times before the head becomes firmly engaged in the upper strait. But should the operator not have succeeded before the waters are discharged, it is not safe nor of any use to persevere in the operation. After the rupture of the amniotic bag, the child must be turned by the feet, or on the head, by internal manipulations. Sometimes it is possible to effect a rectification of the presentation, by a proper posture of the mother on that side where the head of the child is situated, while a pillow is placed underneath the latter. This may be attempted even before labor has begun, as some authors, and especially Dr. Mattei, recommend. The most suitable time for the performance of the operation, as detailed above, is from the beginning of labor to perfect dilatation of the os. The operation has been attempted in the last weeks of pregnancy; but as the pains themselves are a very important aid for the successful performance of the operation, it is not advisable to undertake it at an earlier season. There is no doubt that the position of the fœtus can be changed at any time during the last months of gestation; but it is the general opinion of authors, that a lasting rectification can only be obtained when the operation is performed at the time of the beginning of labor.

The only physical obstacle against a successful operation is an unusual thickness or tension of the abdominal walls, which prevents a clear diagnosis of the presentation. Finally, the womb itself might be by some cause or other too tender to allow of a continued pressing with the hands, or it might be so firmly contracted around the fœtus, owing to a scarcity of liquor amnii, that all our attempts would fail to succeed. But these are coincidences not very often met with, and it is therefore the more to be wondered at that this harmless operation is not generally attempted by obstetricians.

If we consider that by this attempt nothing is lost for future action in another way; if we consider the trifling reaction which it leaves on the system of the mother; if we consider the advantages of a vertex presentation for the life of the child, we feel it our duty to call upon the profession to remember that there is such a thing as turning by external manipulation, and that those who have practised it most often were men the names of whom we only pronounce with feeling of deepest admiration.

